# μ-opioid receptor agonists and psychedelics: pharmacological opportunities and challenges

**DOI:** 10.3389/fphar.2023.1239159

**Published:** 2023-10-11

**Authors:** Leah M. Salinsky, Christina R. Merritt, Joshua C. Zamora, Juliana L. Giacomini, Noelle C. Anastasio, Kathryn A. Cunningham

**Affiliations:** Center for Addiction Sciences and Therapeutics and Department of Pharmacology and Toxicology, John Sealy School of Medicine, University of Texas Medical Branch, Galveston, TX, United States

**Keywords:** opioid use disorder, serotonin, 5-HT_2A_R, MOR, psychedelics

## Abstract

Opioid misuse and opioid-involved overdose deaths are a massive public health problem involving the intertwined misuse of prescription opioids for pain management with the emergence of extremely potent fentanyl derivatives, sold as standalone products or adulterants in counterfeit prescription opioids or heroin. The incidence of repeated opioid overdose events indicates a problematic use pattern consistent with the development of the medical condition of opioid use disorder (**OUD**). Prescription and illicit opioids reduce pain perception by activating µ-opioid receptors (**MOR**) localized to the central nervous system (**CNS**). Dysregulation of meso-corticolimbic circuitry that subserves reward and adaptive behaviors is fundamentally involved in the progressive behavioral changes that promote and are consequent to OUD. Although opioid-induced analgesia and the rewarding effects of abused opioids are primarily mediated through MOR activation, serotonin (**5-HT**) is an important contributor to the pharmacology of opioid abused drugs (including heroin and prescription opioids) and OUD. There is a recent resurgence of interest into psychedelic compounds that act primarily through the 5-HT_2A_ receptor (**5-HT**_**2A**_ **R**) as a new frontier in combatting such diseases (e.g., depression, anxiety, and substance use disorders). Emerging data suggest that the MOR and 5-HT_2A_R crosstalk at the cellular level and within key nodes of OUD circuitry, highlighting a major opportunity for novel pharmacological intervention for OUD. There is an important gap in the preclinical profiling of psychedelic 5-HT_2A_R agonists in OUD models. Further, as these molecules carry risks, additional analyses of the profiles of non-hallucinogenic 5-HT_2A_R agonists and/or 5-HT_2A_R positive allosteric modulators may provide a new pathway for 5-HT_2A_R therapeutics. In this review, we discuss the opportunities and challenges associated with utilizing 5-HT_2A_R agonists as therapeutics for OUD.

## 1 Introduction

Opioid-involved overdose deaths remain at crisis levels in the United States (Ciccarone and Shoptaw, 2022; Ahmad et al., 2023). Initially attributable to overprescription of opioid analgesics for pain control, the overdose crisis is now amplified by deaths involving synthetic opioids in the illicit drug supply (Humphreys et al., 2022; Humphreys and Shover, 2023). Pain management practices evolved to reduce opioid-involved overdoses (Bruera and Del Fabbro, 2018; Friedman et al., 2020), however, a substantial proportion of the population remains at risk for overdose and the evolution to opioid use disorder (**OUD**) (Vowles et al., 2015; Volkow and McLellan, 2016). A debilitating condition with significant health consequences (Hasin et al., 2013), ∼2.7 million people aged 12 or older in the United States were diagnosed with OUD in 2020 (SAMHSA, 2021). The diagnosis of substance use disorders (**SUDs**), including OUD, in the United States is currently based upon the *Diagnostic and Statistical Manual of Mental Disorders-5* (DSM-5) which defines symptoms (e.g., taking more drug than intended, inability to stop, intense craving, withdrawal/tolerance, unsuccessful efforts to control use, etc.) on a continuum from mild (2–3 symptoms) to moderate (4–5 symptoms) to severe (6–11 symptoms) (Hasin et al., 2013). The *International Statistical Classification of Diseases and Related Health Problems-10* (ICD-10) criteria align in general with DSM-5 (Hasin et al., 2013). OUD may develop at opioid doses employed in prescription pain management (McAuliffe, 2013; Minozzi et al., 2013; Campbell et al., 2015; Degenhardt et al., 2021), although the burden of prescription OUD in pain patients is difficult to estimate (Guillou-Landreat et al., 2021; Hasin et al., 2022; Sullivan and Ballantyne, 2022).

The multitude of opioid compounds include opioid poppy alkaloids (e.g., morphine), semi-synthetic (e.g., heroin) and fully synthetic opioids (e.g., fentanyl and fentanyl analogs) which bind to the µ-opioid receptor (**MOR**), a G protein-coupled receptor (**GPCR**). Agonist-induced activation of the MOR mitigates pain and evokes intense and pleasurable euphoria, which in turn contributes to the evolution of OUD (Le Merrer et al., 2009; Baldo, 2016; Higginbotham et al., 2022). The activation of MOR triggers G protein coupling to intracellular signal transduction mechanisms in cells of the peripheral and central nervous system (**CNS**); for instance, MOR-activated Gα_i/o_ inhibits adenylyl cyclase release, calcium- and voltage-gated channels to hyperpolarize and suppress neuronal activity (Higginbotham et al., 2022). The MOR also signals from both membrane and intracellular localizations to bias distinct downstream effector pathways which may differentially drive on- and off-target effects (Stoeber et al., 2018; Pineyro and Nagi, 2021). Chronic opioid exposure results in a myriad of cellular adaptations and plasticity in neural systems that contribute to the development and maintenance of OUD and chronic pain syndromes (Christie et al., 1992; Williams et al., 2001; Christie, 2008; O'Brien, 2009; Puretic and Demarin, 2012; Pitchers et al., 2014).

The MOR is also the target for medications for OUD (**MOUD**) which are effective in treating OUD, managing craving and withdrawal, as well as reducing overdoses (Volkow and McLellan, 2016; Wakeman et al., 2020; Brandt et al., 2023). However, the MOUD medications buprenorphine and methadone are partial and full MOR agonists, respectively, and are subject to abuse, misuse and diversion (Koob and Volkow, 2016; Chilcoat et al., 2019; Strang et al., 2020), prompting the field to mine mechanistic targets outside of the opioid system for novel medications for OUD (Coussens et al., 2019; Marek et al., 2019; Rasmussen et al., 2019). An area of intense interest is the prospect that psychedelics may be harnessed for the treatment of OUD (DiVito and Leger, 2020; Koslowski et al., 2021; Barnett and Weleff, 2022; Urban et al., 2023) and pain conditions (Zia et al., 2023).

Psychedelics include a large group of natural and synthetic compounds (Nichols, 2016) that preferentially bind to the serotonin (5-hydroxytryptamine; **5-HT**) 5-HT_2A_ receptor (**5-HT**_**2A**_ **R**) to alter sensory perceptions and cognitive processes in humans (Quednow et al., 2012; Kometer et al., 2013; Holze et al., 2021a; Becker et al., 2023). Intriguingly, the “classical” psychedelic *d*-lysergic acid diethylamide (**LSD**) was reported to evoke effective analgesia, likely linked to altered pain perception (Kast and Collins, 1964; Ramaekers et al., 2021). Furthermore, analyses of anecdotal and self-reported data support the consideration of psychedelics for treatment of OUD (Savage and McCabe, 1973; Garcia-Romeu et al., 2019; Argento et al., 2022; Jones et al., 2022), and several clinical trials with psilocybin are ongoing for OUD (e.g., NCT0416066, NCT0542029, NCT06005662) and pain conditions (e.g., NCT06001749, NCT05224336, NCT05068791). MOR function is fundamentally integrated with 5-HT neurotransmission (Tao et al., 1998; Tao and Auerbach, 2002b; Tao et al., 2003; Ozdemir, 2017) with a prominent role for the 5-HT_2A_R (Aira et al., 2012; Heijmans et al., 2021; Li et al., 2021; Sierra et al., 2022), from the 5-HT_2_R family (5-HT_2A_R, 5-HT_2B_R, 5-HT_2C_R) (Barnes et al., 2021). As opposed to the MOR, the coupling of the 5-HT_2A_R to Gα_q/11_ heterotrimeric G proteins triggers phosphatidylinositol-4,5-bisphosphate hydrolysis and subsequent release of inositol triphosphate, diacylglycerol, and other effectors (Barnes et al., 2021). Both the 5-HT_2A_R (Barnes et al., 2021) and MOR (Pineyro and Nagi, 2021) interact directly with intracellular β-arrestins to control signaling and membrane trafficking which is driven by a diversity of cellular signaling factors. The MOR and 5-HT_2A_R co-express in individual neurons that mediate aspects of nociception and OUD processes (Lopez-Gimenez et al., 2008; Aira et al., 2012). Co-activation of the 5-HT_2A_R in heterologous cells is permissive in evoking MOR cellular localization and function (Lopez-Gimenez et al., 2008), actions which may contribute to 5-HT-mediated outcomes upon opioid exposure *in vivo* (Tao et al., 1998; Harris and Aston-Jones, 2001; Singh et al., 2003).

Much remains to be learned about cellular and circuit interactions between MOR and 5-HT_2A_R, and the renewed interest in psychedelics for their potential therapeutic efficacy in neuropsychiatric disorders (McClure-Begley and Roth, 2022; Slocum et al., 2022) premises the goal to explore the engagement of 5-HT_2A_R processes in OUD. In the current quest to develop non-opioid medications for OUD, psychedelics may provide value as medication candidates. *The goal of the present review is to explore interactions between opioids and psychedelics as well as the opportunities and challenges for pain and OUD therapeutics*.

## 2 Psychedelic pharmacology

Psychedelics derived from plants and fungi have served significant spiritual and medical purposes since ancient times (Griffiths and Grob, 2010; Doblin et al., 2019; Bouso and Sanchez-Aviles, 2020). Three main structural classes of serotonergic hallucinogens include ergolines (e.g., LSD), tryptamines (e.g., psilocybin, converted by the body to the active molecule psilocin) and phenethylamines [e.g., 2,5-dimethoxy-4-methylamphetamine (**DOM**), 2,5-dimethoxy-4-iodophenyl]-2-aminopropane (**DOI**)]. These compounds share close structural similarity to the endogenous tryptamine 5-HT (Gaddum and Hameed, 1954) which spurred the discovery that 5-HT_2A_R agonist efficacy is a key mechanism of action for this class of compounds to evoke overlapping and powerful subjective effects in humans (Nichols and Walter, 2021), although psychedelic pharmacology is complex and nuanced (Howell and Cunningham, 2015; Castellanos et al., 2020; McClure-Begley and Roth, 2022; Wulff et al., 2023). While the presumed abuse liability and limited medical value historically restricted clinical studies of psychedelics (Henningfield et al., 2022; Hendricks et al., 2023), 5-HT_2A_R neurotransmission was established to mediate hallucinations in schizophrenia and Parkinson’s disease psychosis (Schmidt et al., 1995; Ballanger et al., 2010; Hacksell et al., 2014; Cho et al., 2017). With nanomolar potency as a 5-HT_2A_R inverse agonist/antagonist and selectivity for the 5-HT_2A_R over other GPCRs (Vanover et al., 2006), pimavanserin blocked hallucinations and delusions in refractory schizophrenia (Nasrallah et al., 2019) and Parkinson’s disease patients (Meltzer et al., 2010), and is now approved for treatment for the latter indication (Hacksell et al., 2014; Davis et al., 2021). Although the efficacy of pimavanserin to suppress psychedelic-induced hallucinations is not yet reported, the 5-HT_2_R antagonist ketanserin (Brogden and Sorkin, 1990) reversed the subjective effects (e.g., visual/auditory alterations) of LSD (Preller et al., 2017; Preller et al., 2019; Pokorny et al., 2020; Holze et al., 2021b; Becker et al., 2023) and psilocybin in humans (Quednow et al., 2012; Kometer et al., 2013). It is also important to recognize that the functional network mechanisms underlying psychedelic-vs. disease-evoked hallucinations are distinguishable (Rolland et al., 2014; Leptourgos et al., 2020; Silverstein and Lai, 2021).

Preclinical model systems are essential in linking 5-HT_2A_R functionality with hallucinogenesis, particularly the rodent head twitch response (**HTR**). The HTR is a rapid, rotational head movement evoked by LSD and other hallucinogens which is eliminated in constitutive 5-HT_2A_R knockout mice (Gonzalez-Maeso et al., 2007; Halberstadt and Geyer, 2010). The 5-HT_2A_R antagonist M100907 (MDL100907; volinanserin) exhibits ∼100-fold selectivity at the 5-HT_2A_R versus other monoamine GPCRs (Palfreyman et al., 1993; Kehne et al., 1996; Casey et al., 2022) and blocks psychedelic-evoked HTRs (Canal et al., 2013; Fink et al., 2015; Halberstadt et al., 2020). Importantly, the potency of LSD to evoke the HTR correlates positively with its potency to evoke psychoactivity in humans (Corne and Pickering, 1967), providing a rodent “proxy” for hallucinogenic actions. Direct cortical 5-HT_2A_R activation induces the HTR in rats (Willins and Meltzer, 1997) while the subjective response to psilocybin positively correlated with neocortical 5-HT_2A_R occupancy (Madsen et al., 2019), supporting a critical role of cortical 5-HT_2A_R in psychedelic mechanisms of action.

The case for the role of the 5-HT_2A_R in the mechanisms of action of psychedelics is strong, but we would be remiss to overlook the fact that distinct *in vitro* and *in vivo* profiles are recognized across psychedelic molecules which variably bind to multiple 5-HT receptors, dopamine D_1_ receptor (**D**_**1**_ **R**) and D_2_R, as well as other brain-localized targets (Howell and Cunningham, 2015; Castellanos et al., 2020; McClure-Begley and Roth, 2022; Wulff et al., 2023). For instance, LSD potently binds to human 5-HT_2A_R but also 5-HT_1A_R, 5-HT_2C_R, D_2_R and with some affinity for *α* adrenergic, D_1_R and D_3_R (Rickli et al., 2015; Rickli et al., 2016). LSD exhibits distinct stimulus effects mediated by the 5-HT_2A_R (Cunningham et al., 1985; Nielsen et al., 1985; Cunningham and Appel, 1987; Cunningham and Appel, 1988; Baker, 2018), while lisuride, non-hallucinogenic congener of LSD, only partially substituted for LSD or DOI in drug discrimination analyses and is proposed to engage dopaminergic D_2_R and 5-HT_1A_R signaling (Callahan and Appel, 1990; Marona-Lewicka et al., 2002). Further, the recent discovery that LSD and psilocybin bind to the tropomyosin receptor kinase B receptor for brain-derived neurotrophic factor suggests new complexity to the actions of these intriguing compounds (Moliner et al., 2023).

Some serotonergic hallucinogens exhibit high affinity for both the 5-HT_2A_R and 5-HT_2C_R which colocalize in many of the same brain regions and are reported to interact at the cellular level *in vitro* (Moutkine et al., 2017; Felsing et al., 2018), in rat medial prefrontal cortex (**mPFC**) *ex vivo* (Price et al., 2019), and *in vivo* preclinical models (Pockros et al., 2012; Burton et al., 2013; Cunningham et al., 2013; Anastasio et al., 2015; Bazovkina et al., 2015; Moutkine et al., 2017) to control neural bases for several behaviors (Cunningham and Anastasio, 2014; Howell and Cunningham, 2015). A modulatory role for the 5-HT_2C_R over psychedelic actions is suggested to contribute to some behavioral outcomes in animals (Krebs-Thomson et al., 1998; Smith et al., 2003; Krebs-Thomson et al., 2006), while a functional interaction between the 5-HT_2A_R and 5-HT_2C_R is proposed to mediate *in vivo* effects of psilocybin in mice (Erkizia-Santamaria et al., 2022). Of note, endogenous 5-HT binds to the transmembrane domains that comprise the 5-HT_2A_R orthosteric site which shares ∼80% homology with the 5-HT_2B_R and 5-HT_2C_R (Barnes et al., 2021). Agonist actions at the 5-HT_2B_R cause cardiac valvulopathy and pulmonary hypertension, as seen with the anti-obesity medication fenfluramine (Rothman and Baumann, 2009; Barnes et al., 2021). LSD and psilocin, the active metabolite of psilocybin, exhibit similar affinity at the 5-HT_2A_R and 5-HT_2B_R *in vitro* (Porter et al., 1999), but neither are projected to pose risk for valvulopathy, particularly on acute dosing (Tagen et al., 2023). However, given the critical liability of off-target 5-HT_2B_R agonist actions (Horvath et al., 2004; Hutcheson et al., 2011), current efforts to identify 5-HT_2A_R-selective agonists as novel chemical entities require 5-HT_2B_R affinity and efficacy screening early in molecule discovery (Cao et al., 2022; Chen et al., 2023; Cunningham et al., 2023).

## 3 Abuse liability of MOR agonists vs. psychedelics

The effectiveness of MOR agonists for pain management is complicated by its powerful efficacy to evoke euphoric and pleasurable effects which are at the core of their abuse liability. The FDA guidelines define abuse liability as the probability that a psychoactive drug will sustain patterns of non-medical self-administration, drug-seeking and craving, a triad likely to result in undesirable health consequences (Carter and Griffiths, 2009; Leiderman, 2009; Comer et al., 2012; Calderon et al., 2015). The capacity of a drug to maintain self-administration under experimental conditions in animals aligns well with the likelihood of abuse by humans (Panlilio et al., 2005; Sughondhabirom et al., 2005; Calderon et al., 2015; Calderon et al., 2023). This model system is based upon the operant construct that a psychoactive drug serves as a positive rewarding stimulus which increases the prospect of a behavioral response (e.g., lever press) to gain drug access (Morse and Skinner, 1958). This assay has high predictive validity in assessing the abuse liability of novel drugs and in aligning molecular and neurobiological adaptations associated with repeated drug self-intake in rodents (O'Connor et al., 2011; Belin-Rauscent and Belin, 2012; Cunningham and Anastasio, 2014; Howell and Cunningham, 2015; Neelakantan et al., 2017; Spanagel, 2017; Ezeomah et al., 2020; Merritt et al., 2022) and humans (Jones and Comer, 2013; Stoops, 2022). Self-administration models are widely deployed to identify the primary role for MOR mechanisms in the rewarding properties of opioid agonists (e.g., morphine, heroin, fentanyl) which are prevented by MOR antagonists naloxone or naltrexone (Koob et al., 1984; Beardsley et al., 2004; Leri and Burns, 2005; Vendruscolo et al., 2018). In contrast to MOR agonists, psychedelics [e.g., DOI, DOM, *N*,*N*-dimethyltryptamine (**DMT**), mescaline, psilocybin] exhibit limited reinforcing effects. At best, psychedelics support transient or sporadic drug-taking in self-administration assays (Fantegrossi et al., 2004a; Fantegrossi et al., 2004b; Maguire et al., 2013; Goodwin, 2016; Johnson et al., 2018), although intermittent-access self-administration may be useful to further interrogate psychedelic abuse liability (Goodwin, 2016). Of note, near complete tolerance to psychedelics (e.g., LSD) occurs under several dosing paradigms in humans (Belleville et al., 1956; Rosenberg et al., 1963) and animals (Trulson and Jacobs, 1977; Schlemmer et al., 1986; Buchborn et al., 2015), perhaps accounting for the limited repetitive abuse liability of psychedelics (Belleville et al., 1956).

Only a handful of studies explored the efficacy of psychedelics to alter self-administration of opioid analgesics. The psychedelic DOM attenuated heroin self-administration, resulting in a downward shift of the heroin dose-response curve in one study (Maguire et al., 2013); however, in a more recent study deployed in a sucrose versus drug choice procedure, DOM did not impact fentanyl intake, but did decrease completed choice trials and response rates (Maguire, 2023). Employing a behavioral economics variant of the self-administration paradigm which estimates unique descriptors of opioid demand as a function of its “price,” DOI decreased the motivation to consume fentanyl and total fentanyl consumption in a 5-HT_2A_R-dependent manner (i.e., blocked by M100907) (Martin et al., 2021). While initial research may support the potential usage of psychedelics to reduce the abuse liability and intake of opioids, future studies are necessary to disentangle the key mechanistic facets of psychedelics that impact opioid self-administration. That said, 5-HT_2A_R agonist psychedelics are thought to maintain a large margin of safety and a low level of toxicity (Nichols, 2016; Sakloth et al., 2019), particularly in contrast to opioid agonists (Von Korff et al., 2011; Jeffery et al., 2020; Preuss et al., 2023).

A complementary model to assess abuse liability in keeping with FDA guidelines (Calderon et al., 2015; FDA, 2017; Calderon et al., 2023) is the drug discrimination paradigm which is robust, sensitive and exhibits predictive validity (Appel et al., 1982; McMahon, 2015; Bolin et al., 2018). This assay involves training subjects through differential reinforcement to recognize the stimulus effects of a psychoactive drug versus no (or another) drug. Useful in establishing whether novel CNS-active compounds are predicted to have abuse liability (Soto et al., 2019; Wild et al., 2019; Wold et al., 2020; Stoops, 2022), this assay is an indirect measure of reinforcing efficacy with remarkable pharmacological specificity and utility in assessing *in vivo* pharmacology in humans and animals (Jones and Comer, 2013; McMahon, 2015; Bolin et al., 2018; Stoops, 2022). Drug discrimination analyses established deep knowledge of the neural mechanisms underlying the interoceptive effects of MOR agonists (Beardsley et al., 2004; Meert and Vermeirsch, 2005; Schwienteck et al., 2019; Walker et al., 2021), which are blocked by naloxone or naltrexone (Beardsley et al., 2004; Flynn and France, 2021), albeit larger doses are required to block the long-acting MOR agonist carfentanil (Flynn and France, 2021). Drug discrimination assays also detailed a complex interaction between MOR agonists with kappa and delta opioid receptor agonists and mixed agonists (Picker, 1997; Yadlapalli et al., 2018).

In contrast to the limited psychedelic intake seen in self-administration models, animals readily learn to discriminate the interoceptive effects of hallucinogens (e.g., LSD, DOM, DOI, and others) from no drug (e.g., saline) and cross-substitution occurs across this class of drugs (Appel et al., 1982; Appel and Cunningham, 1986; Fiorella et al., 1995b; a;Baker, 2018; Bolin et al., 2018; Heal et al., 2018). The effective doses of hallucinogens in drug discrimination analyses correlate significantly with their potency to evoke hallucinations in humans, HTRs in rodents as well as affinity as 5-HT_2A_R agonists (Glennon et al., 1983; Sadzot et al., 1989; Nelson et al., 1999; Halberstadt et al., 2020). Notably, for a series of 5-HT_2_R antagonists tested, the dose that blocked 50% of the stimulus effects of LSD correlated significantly with the affinity for the 5-HT_2A_R, but not the 5-HT_2C_R (Nielsen et al., 1985; Fiorella et al., 1995b). This 5-HT_2A_R-dominant mechanism occurs within the 30-min pretreatment timeframe with 5-HT_2A_R antagonists, while a late-stage (90 min) of the stimulus effects of LSD involve dopamine D_2_R properties (Marona-Lewicka et al., 2002). There is conjecture that these time-dependent effects in drug discrimination may align with the transition from the psychedelic effects to aversive aspects of the experience (Marona-Lewicka et al., 2002; Zamberlan et al., 2018). Interestingly, a recent analysis speculated that opioid receptors may be involved in the reported subjective effects of psychedelics (Zamberlan et al., 2018).

## 4 The 5-HT_2A_R as a target for opioid use disorder

**FIGURE 1:**
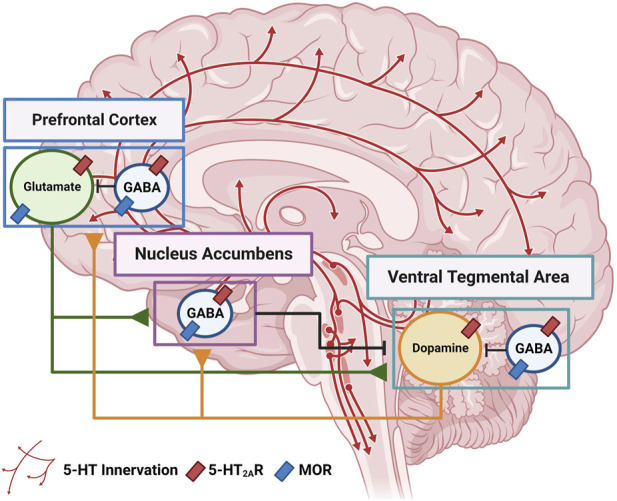
Cellular distribution of 5-HT_2A_R and MOR within meso-corticolimbic circuitry derived primarily from rodent studies. Serotonergic axons from DRN (**red arrows**) innervate the circuit. The VTA consists of locally projecting GABA interneurons (**blue**) and dopaminergic neurons (**orange**) that project to the NAc and PFC. In the PFC, GABAergic interneurons (**blue**) project locally while pyramidal glutamatergic neurons in the PFC (**green**) project to NAc and VTA. The NAc is comprised of GABA medium spiny neurons (**blue**) that project to VTA and other structures not shown. Figure created with BioRender.com.

Opioid signaling, particularly through the MOR, is mechanistically implicated in the rewarding effects of abused opioids (Koob et al., 1984; Negus et al., 1993) as well as pain relief in rodents (King et al., 2011; Okun et al., 2011; Navratilova et al., 2012) and humans (Becerra and Borsook, 2008), effects mediated by meso-corticolimbic circuitry (Strang et al., 2020). These actions in circuitry are ultimately dependent upon the selectivity of opioid compounds for MOR, potency, half-life, pharmacokinetics and specific pharmacological profiles (Vearrier and Grundmann, 2021). With continuing abuse in vulnerable individuals, initially impulsive opioid use evolves to compulsive use which reflects direct and indirect actions and adaptations within neurocircuitry (Madden et al., 1997; Bakhshipour-Rudsari and Karimpour-Vazifehkhorani, 2021; Tolomeo et al., 2021; Peck et al., 2022). The evolution from initial opioid use to diagnosis of OUD involves altered biological homeostasis, rendering behavior increasingly resistant to change, even in the face of the negative consequences (Koob and Volkow, 2016; Strang et al., 2020). An overactive incentive-motivational system centered in ventral tegmental area (**VTA**) dopamine (**DA**) neurons and its neural projections to the nucleus accumbens (**NAc**) serves as the “accelerator” (reward, persistent drug-induced behaviors) (Figure 1). Subregions of the prefrontal cortex (**PFC**) play both direct and subtle regulatory roles as a “behavioral brake” over drug use (inhibitory control, executive function) and is vulnerable to disruption by chronic opioid exposure leading to heightened preoccupation and anticipation of drug acquisition (Ujcikova et al., 2021; Ceceli et al., 2023). A well-characterized role for the 5-HT_2A_R in meso-corticolimbic neurocircuitry serves to illustrate the power and complexity of this system (Figure 1).

A large population (50%–66%) of PFC pyramidal neurons express the 5-HT_2A_R (Blue et al., 1988; Miner et al., 2003; Santana et al., 2004) within layer V pyramidal neurons (Pompeiano et al., 1994; Lopez-Gimenez et al., 2001; Amargos-Bosch et al., 2004; Price et al., 2019) which affords stimulatory glutamate drive to the VTA DA neurons (Vazquez-Borsetti et al., 2009; Vazquez-Borsetti et al., 2011) and NAc (Mocci et al., 2014), indirectly enhancing mesolimbic DA neurotransmission. Intriguingly, PFC axons preferentially contact VTA DA neurons that project back to the mPFC (Carr and Sesack, 2000). Systemic administration or direct infusion of the 5-HT_2A_R agonist and hallucinogen DOI into the medial PFC (**mPFC**) not only evokes the HTR (Willins and Meltzer, 1997), but also increases the rate of firing of VTA DA neurons (Bortolozzi et al., 2005) with both outcomes blocked by the selective 5-HT_2A_R antagonist M100907 (Willins and Meltzer, 1997; Bortolozzi et al., 2005). Intra-mPFC infusion of M100907 blocked DOI-evoked mesocortical DA release in the VTA and DA release in the mPFC of the same rats, supporting the clarification that 5-HT_2A_R activation of corticotegmental glutamate projections synapse on mesocortical DA neurons (Pehek et al., 2006). Also, systemic DOI increased glutamate release in the VTA, which was blocked by intracortical administration of M100907 (Pehek et al., 2006). Thus, the 5-HT_2A_R in PFC projections increases stimulatory drive on the VTA, explaining the observed 5-HT_2A_R-stimulated DA release in the PFC (Bortolozzi et al., 2005; Pehek et al., 2006). Of note, this multi-synaptic template is likely to have additional elements, since approximately 50% of the 5-HT_2A_R-expressing pyramidal neurons that synapse in the VTA also synapse in the dorsal raphe nucleus (**DRN**), the major contributor to serotonergic innervation of PFC (Vazquez-Borsetti et al., 2009) with broad opportunities for modified 5-HT_2A_R control of meso-corticolimbic pathways involved in opioid actions. Further, activation of the 5-HT_2A_R is essential for induction of cortical neuroplasticity, giving rise to the hypothesis that 5-HT_2A_R agonists hold promise to “liberate” cortical activity as a therapeutic approach (Ly et al., 2018). Future studies should explore the nature of “corrective” neuroplasticity (as may occur with psychedelics) vs. “deranged” neuroplasticity (noted as decreased function of normal reward and cognitive function in OUD) to understand vulnerability to continued drug use and relapse vulnerability (Koob and Le Moal, 2005).

Opioid and 5-HT_2A_R biology intersect at multiple circuit and cellular levels (Barnes et al., 2021; Heijmans et al., 2021; Sasaki et al., 2021). Systemic administration of the MOR agonists morphine and fentanyl acutely raises extracellular 5-HT levels in the DRN and areas of the forebrain that receive innervation from 5-HT DRN efferents (i.e., PFC, striatum, amygdala, etc.) (Tao and Auerbach, 1995; 2002a; Tao et al., 2003) (Figure 1). Interestingly, this effect wanes upon repeated exposure to opioid agonists indicating a reduced responsivity of 5-HT signaling with chronic opioid use (Tao et al., 1998). Extracellular 5-HT in the raphe is also significantly diminished during opioid withdrawal which likely contributes to abstinence evoked phenomena (Tao et al., 1998). Further, functional crosstalk between the MOR and 5-HT_2A_R was suggested to regulate opioid-mediated abuse liability (Marek, 2003; Lopez-Gimenez et al., 2008; Mohammadi et al., 2016; Pang et al., 2016; Neelakantan et al., 2017; Sierra et al., 2022). Morphine and other opioids suppress LSD-evoked HTRs, most likely through 5-HT_2A_R agonist actions (Corne et al., 1963; Vetulani et al., 1980). The selective 5-HT_2A_R antagonist M100907 augments the antinociceptive effects of the MOR agonist oxycodone in a sex-specific manner in mice supporting a role for 5-HT_2A_R mechanisms in MOR evoked analgesia (Sierra et al., 2022). The psychedelics DOM and quipazine (de la Fuente Revenga et al., 2021) decreased the discriminative stimulus effects of morphine in monkeys in a 5-HT_2A_R-dependent manner (Li et al., 2011). Nonetheless, the exact mechanistic nature of this interaction between 5-HT_2A_R and MOR systems is difficult to disentangle based upon current knowledge, but we propose the convergent effects of the 5-HT_2A_R and opioid systems may occur at the level of the mPFC (Figure 1) (Willins and Meltzer, 1997).

Signaling through MOR VTA and NAc are well studied (Trigo et al., 2010; Fields and Margolis, 2015), and the MOR also localizes in high density to the PFC (Tempel and Zukin, 1987; Mansour et al., 1988; Mansour et al., 1995) (Figure 1) which is highlighted as a “hot spot” for dysregulated motivational function and opioid-mediated behaviors (Baldo, 2016). The MOR localizes in γ-aminobutyric acid (**GABA**) interneurons in the neocortex and is proposed to disinhibit glutamate-driven outflow to its terminal fields (Taki et al., 2000; Ferezou et al., 2007). A subset of cortical glutamate neurons is also reported to express MOR (Birdsong et al., 2019; Zhang et al., 2020; Du et al., 2022). Opportunistically, the 5-HT_2A_R localizes within these key regions (Figure 1) (Liu et al., 2014; Howell and Cunningham, 2015; Browne et al., 2019; Cunningham et al., 2020; Nagai et al., 2020). Intriguingly, repeated morphine treatment of rats elevates 5-HT_2A_R protein levels within the PFC as assessed *ex vivo* (Pang et al., 2016), while altered 5-HT_2A_R-mediated Gα_q/11_ activation was observed in the dorsolateral PFC dissected from *postmortem* brains of OUD patients (Odagaki et al., 2021), suggesting that the 5-HT_2A_R in PFC plays a role in the modulation of processes involved in OUD.

Cellular colocalization of 5-HT_2A_R and MOR transcripts was observed in layer V of rat neocortex (Lopez-Gimenez et al., 2008), although the resident cell type remains to be described. MOR agonists suppress 5-HT_2A_R agonist-mediated excitation of PFC pyramidal neurons (Marek and Aghajanian, 1998). Along these lines, in the presence of 5-HT_2A_R expression, upregulation of MOR levels was observed, and co-addition of 5-HT plus morphine resulted in rapid desensitization, internalization and downregulation of MOR in cellular models; these effects were not observed in the absence of 5-HT_2A_R transfection (Lopez-Gimenez et al., 2008). As morphine-induced MOR desensitization was demonstrated to maintain potent analgesic efficacy without leading to antinociceptive tolerance (Kim et al., 2008), co-activation of 5-HT_2A_R and MOR may provide an opportunity for opioid-sparing therapy for conditions of chronic opioid pain management or to reduce the likelihood of OUD development. Taken together, these behavioral and cellular observations putatively support a cooperative MOR and 5-HT_2A_R regulation of outflow of the PFC (Marek and Aghajanian, 1998) to control output of OUD neurocircuitry, and premise future studies to further clarify this hypothesis.

## 5 Novel perspectives in 5-HT_2A_R molecule discovery

Largely based on the historical bias that all psychedelics evoke hallucinations and have abuse potential (and remain in Schedule I), there is an important gap in the preclinical profiling of 5-HT_2A_R agonists in OUD models. New chemical biology initiatives are underway to identify novel 5-HT_2A_R agonist-targeted molecules with distinctive profiles, perhaps lacking hallucinogenic effects, as seen with structural congeners the hallucinogen LSD and non-hallucinogen lisuride (White and Appel, 1982; White, 1986; Gonzalez-Maeso et al., 2003). In particular, there is intense investment in characterizing the promise of hallucinogenic and non-hallucinogenic 5-HT_2A_R agonists for medications development for OUD and other SUDs (DiVito and Leger, 2020; Koslowski et al., 2021; Barnett and Weleff, 2022; Jones and Nock, 2022; Urban et al., 2023). One such effort focused on ibogaine, an indole alkaloid derived from the evergreen shrub *Tabernanthe iboga* (Jenks, 2002). Ibogaine is a molecule with affinity for opioid receptors which is reported to decrease opioid withdrawal symptoms (Parke et al., 2002; Mash, 2023) and alleviate drug cravings (Mash et al., 2016; Mash et al., 2018; Noller et al., 2018; Mash, 2023). The hallucinogen ibogaine has a complex mechanism of action (Mash et al., 1995a; Mash et al., 1995b) and an unacceptable safety profile (Koenig et al., 2014; Thurner et al., 2014), but recently served as a promising starting point for new chemical entities (Cameron et al., 2021). The synthesis of ibogaine analogs resulted in identification of tabernanthalog (**TBG**) which exhibits a better preclinical safety profile than ibogaine (Cameron et al., 2021). The TBG binding profile is, however, complex as multiple receptor systems are involved in its pharmacology, including the 5-HT_2A_R, 5-HT_2C_R as well as other monoamine receptors, the 5-HT transporter and monoamine oxidase (Cameron et al., 2021). This unique molecule did not evoke HTRs in rodents, suggesting the possibility that TBG serves an example of a “non-hallucinogenic” derivative which is effective in reducing alcohol and heroin intake in rodents, but not sucrose ingestion, effects blocked by ketanserin (Cameron et al., 2021; Heinsbroek et al., 2023).

An historically interesting compound is BOL-148 (2-bromo-LSD) (Troxler and Hofmann, 1957; Lewis et al., 2023) which was initially identified in the 1950s as lacking hallucinogenic effects (Turner et al., 1959; Karst et al., 2010). BOL-148, long recognized for its inability to evoke HTRs (Corne and Pickering, 1967), blocked the psychological effects of LSD in a small human study (Ginzel and Mayer-Gross, 1956). More recent studies validated that BOL-148 lacks efficacy to evoke HTRs but does exhibit an antidepressant-like profile in mice, and 5-HT_2A_R-dependent neuronal plasticity (Lewis et al., 2023). An additional molecule, Ariadne (4-methyl-2,5-dimethoxy-alpha-ethylphenethylamine) did not evoke classical psychedelic effects in humans (Shulgin and Shulgin, 1991) nor the HTR in mice (Cunningham et al., 2023), but drug discrimination analyses demonstrated substitution for the empathogen 3,4-methylenedioxymethamphetamine (Glennon, 1993) and LSD (Winter, 1980). Thus, Ariadne exhibits an interesting behavioral profile as a unique non-hallucinogenic molecule possibly distinguished from classical hallucinogens at the level of restricted 5-HT_2A_R signaling efficacy (Cunningham et al., 2023).

These advances are key steps toward our future understanding of 5-HT_2A_R signaling specificity as well as the requirements for maximizing psychedelics for therapeutic efficacy and rationally designing such analogs (Ly et al., 2018; Olson et al., 2018; Cameron et al., 2021; Dong et al., 2021; Cao et al., 2022; Cunningham et al., 2023). Additionally, molecular studies of ligand-activated GPCRs and GPCR-transducer complexes are expanding our understanding of how 5-HT_2A_R activation leads to biased and unbiased signal transduction (Hilger et al., 2018; Kim et al., 2020). As the science of 5-HT_2A_R molecular signaling advances to define hallucinogenic versus non-hallucinogenic 5-HT_2A_R agonists, future studies will allow interrogation of how 5-HT_2A_R agonists with distinct profiles of biased signaling efficacy may impact opioid-evoked behaviors and their relative propensity to correct opioid-mediated neuronal dysfunction.

Selective 5-HT_2A_R activation may be useful in combating impaired serotonergic control that contributes to vulnerability to pain conditions and OUD. As noted, selective agonist targeting of the 5-HT_2A_R and/or 5-HT_2C_R is challenged by the high sequence homology with the 5-HT_2B_R at the 5-HT orthosteric binding pocket at which repeated activation can cause valvulopathy and pulmonary hypertension (Fitzgerald et al., 2000). While activation of the 5-HT_2C_R may be a benefit for pain management and/or treatment of OUD (Nakai et al., 2010; Neelakantan et al., 2017; Ma et al., 2019; Rasmussen et al., 2019; Higgins et al., 2020), there are on-target side effects such as hypophagia and anhedonia (Lam et al., 2008; Yamamoto et al., 2017) as is the case for 5-HT_2A_R agonists (hallucinations) that may limit their clinical practicality (Shalit et al., 2019; Olson, 2021). In addition to direct agonist actions at GPCRs, allosteric modulation is an alternate strategy to design ligands that do not directly engage the orthosteric binding pocket, but rather modulate the function of the GPCR. A positive allosteric modulator (**PAM**) can increase receptor functional responses and/or the intrinsic affinity for the orthosteric ligand through binding to sites on GPCRs that are topographically distinct from the orthosteric site targeted by endogenous (i.e., 5-HT) and synthetic agonists (Lindsley et al., 2016; Wold et al., 2019a). Several medicinal chemistry campaigns reported 5-HT_2C_R PAMs with pharmacological profiles different from agonists (Garcia-Carceles et al., 2017; Wold et al., 2019b; Singh et al., 2019; Wild et al., 2019; Wold et al., 2020; Chen et al., 2023). In this light, oleamide is an intriguing example of an endogenous lipid in mammals (Arafat et al., 1989; Cravatt et al., 1995) that controls behavior (Hedlund et al., 2003; Soria-Gomez et al., 2010; Prospero-Garcia et al., 2016; Mendez-Diaz et al., 2019) and activates 5-HT_2A_R signaling (Cao et al., 2022), but non-selectively (Thomas et al., 1997; Boger et al., 1998; Cheer et al., 1999; Hedlund et al., 1999; Fedorova et al., 2001). A recent synthetic campaign resulted in a series of oleamide-based analogues characterized as selective 5-HT_2C_R PAMs or dual 5-HT_2C_R/5-HT_2A_R PAMs (Chen et al., 2023), as seen with another the natural product derivative PNU-69176E (Im et al., 2003; Ding et al., 2012). In the process, CTW0404 and CTW0419 were identified as potential 5-HT_2A_R PAMs which exhibit distinct pharmacological profiles relative to 5-HT_2A_R agonists (WO2023023287A1) (Zhou et al., 2023). These new discoveries allow for future studies to explore the possibility that 5-HT_2A_R PAMs may be an alternative strategy to engage a subset of 5-HT_2A_R-mediated biological actions distinct from psychedelic 5-HT_2A_R agonists.

## 6 Conclusion

Medications that are FDA-approved for OUD are available, however even when utilized, OUD patients maintain a high propensity for relapse and overdose death (Brandt et al., 2023) and suffer adverse side effects on current MOUD (FDA, 2018). Here, we reviewed evidence for 5-HT_2A_R agonists as novel therapeutics for OUD and propose potential next steps in the elucidation of the complex interactions occurring between 5-HT_2A_R and MOR systems. The behavioral evidence implicates the potential of functional MOR and 5-HT_2A_R interactions in regions controlling opioid-evoked behavioral outcomes and MOR and 5-HT_2A_R are known to colocalize within nodes of OUD circuitry. However, the degree to which cellular and signaling dynamics mediate crosstalk between the MOR and 5-HT_2A_R in the mPFC and how these may be modulated by opioids or exploited by psychedelics remains unresolved. Notably, we focus here on MOR to the exclusion of kappa, delta and nociceptin receptors which are involved in pain (Chu Sin Chung and Kieffer, 2013; Zaveri, 2016; Snyder et al., 2018; Quirion et al., 2020) and OUD (Butelman et al., 2012; Chu Sin Chung and Kieffer, 2013; Valentino and Volkow, 2018; Ubaldi et al., 2021) and could play a role in the psychomimetic effects of psychedelics (Clark and Abi-Dargham, 2019). Further, while classical psychedelics that act as 5-HT_2A_R agonists are showing promise for treatment of facets of OUD, these molecules carry risks (abuse, side effects) and may not be a viable option for many patients. Thus, further analyses of the profiles of non-hallucinogenic 5-HT_2A_R agonists and/or 5-HT_2A_R PAMs provides a new frontier to further characterize the therapeutic potential of the 5-HT_2A_R. Finally, as discussed above psychedelics exhibit a promiscuous pharmacology (e.g., 5-HT_2C_R affinity and efficacy) and how these “off-target” effects contribute to therapeutic efficacy is an important question for the field at large as is a greater knowledge of the interactions between psychedelics and the opioid system.

## Abbreviations

5-HT, serotonin; 5-HT2AR, 5-HT2A receptor; BOL-148, 2-bromo-LSD; CNS, central nervous system; D1R, dopamine 1 receptor; D2R, dopamine 2 receptor; DA, dopamine; DMT, N,N-dimethyltryptamine; DOI, 1-[2,5-dimethoxy-4-iodophenyl]-2-aminopropane; DOM, 1-(2,5-dimethoxy-4-methylphenyl)-2-aminopropane; DRN, dorsal raphe nucleus; GABA, γ-aminobutyric acid; GPCR, G protein-coupled receptor; HTR, head twitch response; LSD, d-lysergic acid diethylamide; MOR, µ-opioid receptor; MOUD, medication for opioid use disorder; mPFC, medial prefrontal cortex; NAc, nucleus accumbens; OUD, opioid use disorder; PFC, prefrontal cortex; TBG, tabernanthalog; VTA, ventral tegmental area.
